# Decreased Coherent Motion Discrimination in Autism Spectrum Disorder: The Role of Attentional Zoom-Out Deficit

**DOI:** 10.1371/journal.pone.0049019

**Published:** 2012-11-06

**Authors:** Luca Ronconi, Simone Gori, Milena Ruffino, Sandro Franceschini, Barbara Urbani, Massimo Molteni, Andrea Facoetti

**Affiliations:** 1 Developmental and Cognitive Neuroscience Lab, Department of General Psychology, University of Padua, Padua, Italy; 2 Developmental Neuropsychology Unit, Scientific Institute “Eugenio Medea”, Bosisio Parini, Italy; University of Regensburg, Germany

## Abstract

Autism spectrum disorder (ASD) has been associated with decreased coherent dot motion (CDM) performance, a task that measures magnocellular sensitivity as well as fronto-parietal attentional integration processing. In order to clarify the role of spatial attention in CDM tasks, we measured the perception of coherently moving dots displayed in the central or peripheral visual field in ASD and typically developing children. A dorsal-stream deficit in children with ASD should predict a generally poorer performance in both conditions. In our study, however, we show that in children with ASD, CDM perception was selectively impaired in the central condition. In addition, in the ASD group, CDM efficiency was correlated to the ability to zoom out the attentional focus. Importantly, autism symptoms severity was related to both the CDM and attentional zooming-out impairment. These findings suggest that a dysfunction in the attentional network might help to explain decreased CDM discrimination as well as the “core” social cognition deficits of ASD.

## Introduction

Individuals with autism spectrum disorder (ASD) show abnormalities in communication and social interaction, as well as markedly restricted interests and stereotyped behaviors [1]. Several studies have reported abnormalities in basic visual perception in ASD [2], [3]. In particular, a detail-oriented perception could be related to the “core” deficits in the social domain, including face processing [4] and emotion recognition through observation of body movements [5].

In the human visual system, the retina transmits information to the lateral geniculate nucleus and then to the primary visual cortex via two main separate pathways: the magnocellular and parvocellular streams [6]. In the extra-striate cortical regions the magnocellular cells provide the principal input to the dorsal stream leading to the dorsolateral occipital cortex and posterior parietal lobe regions [6]. The magnocellular-dorsal (M-D) stream responds to rapidly changing stimuli such as flicker and motion [7].

Braddick and colleagues [8] reported a great deal of evidence suggesting that later development of the M-D stream provides more opportunity for neurodevelopmental abnormalities (i.e., the dorsal stream vulnerability hypothesis). They suggest that this vulnerability is not specific to one particular condition, but rather, it is characteristic of many developmental disorders (e.g., ASD, Developmental Dyslexia and Williams Syndrome).

The coherent dot motion (CDM) paradigm [9] has often been employed to investigate the integrity of M-D stream in several neurodevelopmental disorders (see [10] for a review). In the common version of CDM tasks, a variable proportion of dots (i.e. the signal) move coherently, while the remaining dots (i.e., the noise) move in random directions, at the same speed. Participants are required to make a judgment about the direction of the moving dots, and accuracy increases as a function of the dot proportion moving in the same direction.

Several studies have shown that individuals with ASD require about 10% more coherent motion to correctly report the direction in the CDM paradigm ([11], [12], but see also [13], [14]) or in other similar tasks [15]. Some authors, however, consider the M-D stream vulnerability hypothesis inappropriate for ASD [10], [16], [17]. For example, Pellicano and colleagues [16] found dissociation in individuals with ASD when evaluating different M-D processing stages. They administered both the CDM (testing the high-level stage of the M-D stream) and the flicker contrast task (testing a lower-level stage of the M-D stream) to participants with ASD. Children with ASD were less able to perceive the CDM than typically developing children. However, children with ASD performed no differently from comparison children on the flicker contrast task. Similar findings have been reported by Bertone and colleagues [18]. These results support the hypothesis of a general impairment in the signal integration processing, rather than a specific M-D stream deficit.

Another hypothesis that could explain the poor performance of children with ASD on the CDM task is the perceptual noise exclusion deficit. For example, Sperling and colleagues [19] found that children with developmental dyslexia performed poorly in the CDM task. These authors suggested that their results could be interpreted as a noise exclusion deficit (see also [20]). Signal enhancement and noise exclusion are two important mechanisms to improve perception [19]. Signal enhancement involves maintaining signal integrity during processing, while noise exclusion involves optimizing the perceptual filter. A noise exclusion deficit could also be suitable for the decreased CDM performance shown by children with ASD.

Another aspect that deserves consideration as a possible influence on performance in CDM tasks is spatial attention efficiency. Indeed, neuroimaging and neurophysiological studies suggest a possible top-down role for the fronto-parietal attentional mechanisms in the integration of spatio-temporal information [21]–[23]. For example, Liu and colleagues showed how efficient spatial attention orienting could improve performance in a CDM task [24]. However, the attentional focus is not only oriented towards a specific location, but also has to be adjusted in size. This ability, causally linked with the right frontal eye fields [25], allows processing visual stimuli from a narrow (zoom-in) or a broad (zoom-out) visual region [26], [27], [28].

ASD has been repeatedly associated with different types of dysfunctions in spatial attention [28]–[30]. A recent study by Ronconi and colleagues [30] investigated the efficiency of attentional zooming mechanisms (zoom-out and zoom-in) in children with ASD. Results support the hypothesis of a specific zoom-out attentional impairment in ASD, suggesting that in observers with ASD attentional resources appear to be rigidly allocated in a narrow region of the visual field [30]. Mann and Walker [29] reported similar findings, employing a paradigm that required participants to decide which of two pairs of crosshairs was the longest. ASD participants were less able to make this judgment than the comparison group when the previous pair of cross-hairs was smaller than the one to be judged. The authors argued that individuals with ASD experienced difficulty in zooming out the attentional spotlight, in agreement with findings by Ronconi and colleagues [30].

Thus, if the attentional zoom-out mechanism is specifically impaired, and consequently, the attentional resources are rigidly allocated in a narrow region of the visual field, the global spatio-temporal integration of the coherently moving dots could be inefficient because the outer portion of dot display simply exceeds the size of the attentional focus.

**Figure 1:**
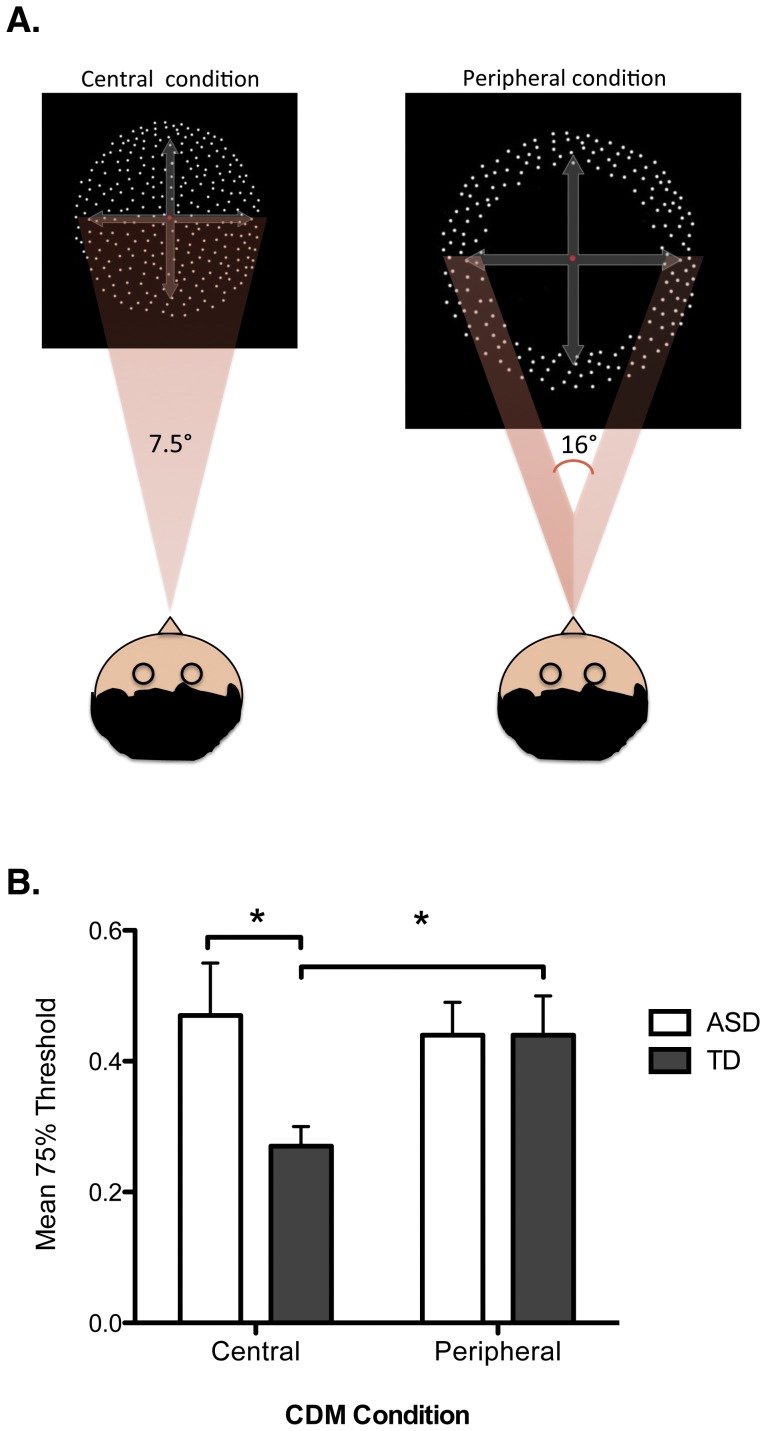
Design and results of the Coherent Dot Motion (CDM) task. Panel A: Schematic representation of central and peripheral condition of the CDM task used in the present study. Panel B: Graph shows mean threshold as a function of group (ASD and TD) and condition (central and peripheral CDM). Error bars represent the standard error of the mean. * represents a significant difference revealed by planned comparisons (p<.05).

In this study, we modified the classic CDM paradigm in order to test whether children with ASD demonstrate a general M-D stream vulnerability, a perceptual noise exclusion deficit, or inability to integrate visual information because of their problem in zooming out the attentional focus. CDM perception was measured under two different conditions: (i) In the central condition, the moving dots appeared inside a circle in the central (foveal and para-foveal) portion of the visual field, and (ii) in the peripheral condition, the moving dots were included within an annulus (empty central portion); namely, the motion perception was measured only in the periphery (see Figure 1A). Since the motion integration required for the CDM task is more efficient in the central visual field [31]–[33], we expected better performance in the central than in the peripheral condition for the typically developing group.

According to a general M-D stream vulnerability [8], one would expect that children with ASD would perform poorer in both the central and peripheral conditions, since the M-D deficit should not affect the two portions of the visual field differently. The same expectation is valid for the perceptual noise exclusion deficit hypothesis [19], since the signal-noise ratio was equal in central and peripheral conditions.

On the other hand, a zoom-out attentional deficit in children with ASD should lead to impairment mainly in the central condition. During the central condition, the narrow attentional focus characterizing the children with ASD would select a small central portion of the dots display, excluding the outermost part. The presence of task-relevant information inside the attentional focus (i.e. moving dots) should, indeed, amplify the difficult in zooming out the focus of attention [29], [30]. The result of this inappropriate attentional processing will diminish available information regarding the coherently moving dots. On the other hand, in the peripheral condition, participants with ASD should be forced to enlarge their attentional focus because of the complete absence of task-relevant information (i.e., moving dots) in the central portion of the visual field, helping the motion integration process. The two experimental conditions, central and peripheral, were performed in separate blocks, to avoid a rapid switch between a narrowed or broadened attentional focus that seems to be impaired in children with ASD [30]. One of our interests was to test if this difficulty in zooming out the attentional focus also persisted in the condition where the information was present only in the periphery, and the time taken to adapt the focus size was not an issue.

## Methods

### Participants

Twenty-two children took part in the present study (see Table 1 for descriptive statistics). All participants of the ASD group (11 children) were selected following these criteria: (i) full scale IQ >70 as measured by the Italian version of the Wechsler Intelligence Scale for Children-Revised [34]; (ii) absence of gross behavioral problems; (iii) normal or corrected-to-normal vision and hearing; (iv) absence of pharmacological therapy; and (v) absence of attention deficit hyperactivity disorder on the basis of DSM-IV criteria [1]. ASD participants were recruited at the Developmental Neuropsychology Unit, “E. Medea” Research Hospital. Diagnosis of ASD was made by licensed clinicians with experience in the assessment of ASD in terms of DSM-IV diagnostic criteria and the autism diagnostic observation scale (ADOS, [35]). For the control group, eleven typically developing (TD) children were randomly sampled from several Padua public schools. The parents reported no prior history of psychiatric disorder in their TD children. Both groups were matched for chronological age (t_(20)_ = .55, p = .59). The cognitive level of TD children was estimated with two verbal (vocabulary and similarities) and two performance (block design and pictures completion) subtests of the WISC-R [34]. ASD and TD group differed only in the vocabulary subtest (t_(20)_ = −2.92, p<.05). Informed written consent was obtained from each child and his or her parents. The ethics committee of the “E. Medea” Hospital review board specifically approved this study. The entire investigation process was conducted according to the principles expressed in the Declaration of Helsinki.

**Table 1:** Descriptive statistics for autism spectrum disorder (ASD) and typically developing (TD) groups.

	ASD (n = 11)	TD (n = 11)
**Age (SD, range)**	13.73 (2.69, 9–18)	13.09 (2.70, 11–18)
**Male∶female ratio**	11∶0	8∶3
**PIQ (SD, range)**	101.91 (14.12, 80–129)	-
**VIQ (SD, range)**	99.09 (13.96, 79–122)	-
**TIQ (SD, range)**	100.55 (14.88, 79–130)	-
**Vocabulary (SD, range)**	9.18 (3.31, 5–14)	12.57 (1.96, 10–16)
**Similarities (SD, range)**	10.82 (2.14, 8–14)	12.43 (1.47, 10–16)
**Picture Completion (SD, range)**	10.64 (2.94, 8–17)	10.83 (0.94, 9–12)
**Block Design (SD, range)**	10.81 (3.57, 4–16)	12.17 (1.94, 8–15)
**ADOS Communication (SD, range)**	3.41 (1.11, 1–5)	-
**ADOS Social Interaction (SD, range)**	5.39 (2.46, 1–8)	-
**ADOS Total (SD, range)**	8.8 (3.15, 2–12)	-

PIQ = performance intelligence quotient, VIQ = verbal intelligence quotient, TIQ = total intelligence quotient. Vocabulary, similarities, Picture completion, and block design are subtests from WISC-R [34].

### Central and peripheral CDM task

Participants were seated in a dimly lit room in front of a 15-in. CRT monitor (screen resolution 1024×768/60 Hz, with 0.3 mm of pixel size). The fixation point was a red dot in the center of the screen. After 500 ms, white dots, subtending a visual angle of 0.05 deg in the central condition and of 0.15 deg in the peripheral condition, appeared on a black background (Michelson contrast between dots and background was 98%). In the periphery, larger dots were employed to reduce the effect of the cortical magnification factor [36], characterized by a larger representation in the visual cortex of the foveal and parafoveal retinal portions compared to peripheral regions. The dots size was scaled according to the procedure elucidated in the work by Carrasco and Frieder ([37], based on equations originally introduced by Rovamo and Virsu [38], [39]). In the central condition, a circle of 7.5 deg of diameter contained all dots. In the peripheral condition dots were within an annulus obtained with an outward circle of 21 deg and an inner circle of 16 deg of diameter. This display resulted in a central empty circle where no dot was presented (see Figure 1A). The number of dots was approximately 17 per deg^2^ at each frame. Frame duration was 16.7 ms. The dots density remained constant through the trial. The dots were constructed in 3 sets plotted in sequence. For each set, the probability that a dot moved in a specific direction as opposed to randomly is given by the coherence value. Consequently, this routine generated dots with a limited lifetime of 3 frames (corresponding to ∼50 ms) after that the dot was randomly relocated in another position inside the display. Dots' speed was 12 deg/sec in both conditions. The CDM display duration was 300 ms, which is around the lower limit to allow global motion integration [40]. Stimuli were presented briefly enough to prevent saccades to the dots display or pursuit attempts. Participants were asked to discriminate the direction of dot movement (upward, downward, leftward or rightward), and only response accuracy was collected (we specified to the participants that response speed was not relevant). All the parameters of our CDM task are within a typical range found in previous literature [41]. There were four levels of coherence, randomly intermixed, at which dots could move (10, 20, 30 and 80%). These values were chosen on the basis of pilot observations. The experimental session consisted of 160 trials, 80 in the central condition and 80 in the peripheral condition (20 trials for each coherence level). The two blocked conditions were counter-balanced across participants.

### Attentional zooming task

**Figure 2:**
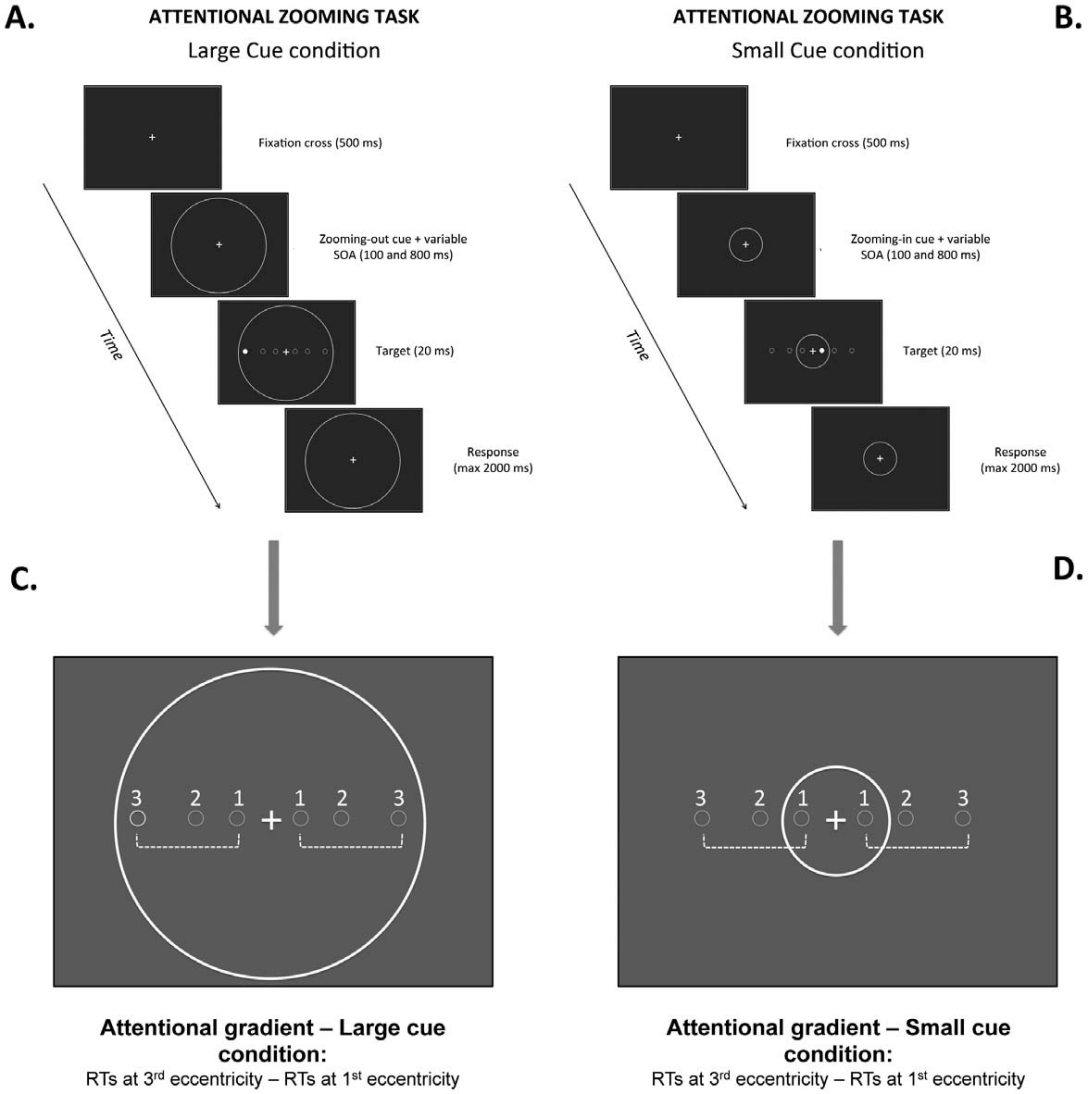
Design of the attentional zooming task. Panel A (large cue condition) and B (small cue condition) show the two types of trials in the attentional zooming task employed in Ronconi and colleagues. [30]. Panel C and D reported a schematic illustration of how the attentional gradient was computed (i.e., a measure of the zoom-in/zoom-out mechanism). The gradient effect in the large cue represents a measure of the ability to enlarge the attentional focus (zoom-out), whereas the gradient effect in the small cue represents a measure of the ability to narrow the attentional focus (zoom-in).

In Ronconi and colleagues [30], we measured the attentional zooming combining the gradient [42] and cue-size effect [25], [27], in order to measure children with ASD's ability to adapt the size of the attentional focus compared to TD children. In the present study, participants performed both the central and peripheral CDM task and the attentional zooming task. Although an exhaustive explanation of the method and the results of the attentional zooming task can be seen in the previous paper [30], we summarize the method here to help readers understand the task. The attentional zooming experiment is a simple detection task, consisting of two cue-size conditions (see Figure 2A and 2B). The small cue condition was characterized by a circle with a diameter of 8 deg presented concentrically to the fixation point, while the large cue condition consisted of a circle with a diameter of 25 deg. The target stimulus was a dot of 0.5 deg, which could appear at one of three possible distances from the fixation point on the horizontal axis (i.e., 2, 6 and 12 deg, named eccentricity 1, eccentricity 2 and eccentricity 3, respectively). In the large cue condition, the target was always displayed inside the focusing cue (see Figure 2A). In contrast, in the small cue condition, at eccentricity 1, the target was displayed inside the focusing cue, whereas at eccentricity 2 and 3, it fell outside (see Figure 2B). Each trial started with the onset of the fixation point. After 500 ms, a non-informative small or large zooming cue was presented. A variable cue-target stimulus onset asynchrony (SOA) of 100 or 800 ms was employed before the target presentation (duration = 16.7 ms). Participants were instructed to press the space bar on the keyboard as fast as possible at target onset. Catch trials, in which the stimulus was not presented and the participant did not have to respond, were intermixed with response trials. Reaction times (RTs) were recorded relative to the onset of the stimulus. We predict that typically developing participants will be able to zoom their attention in, generating a gradient effect only when a small cue anticipates the target onset. On the other hand, with a large cue, they should be able to zoom out their attention, nullifying the gradient effect of the target eccentricity. This prediction should be valid only at the shorter stimulus onset asynchrony (SOA, i.e., 100 ms) because, when a longer SOA is employed (i.e., 800 ms), the time between the cue and the target will be too long to sustain the zoom in of attentional focus [43]. Thus, our prediction is that if the zoom-out attentional mechanism is specifically impaired in children with ASD, these children will show an abnormal gradient effect in the large focusing cue only at short cue-target SOA.

## Results

### Central and peripheral CDM task

Mean accuracy data at the different coherence levels were fitted for each subject with a logistic function. The upper bound was set to 1 and the lower bound to y0 = 0; y = 0 means that the correct dot direction was never properly signaled; y = 1 indicates that at a given dot coherence motion, the correct dot direction was always reported. The only free parameters of the function are therefore b (the function slope) and t (the threshold at 50% of correct motion discrimination). The resulting logistic function (the same as used previously for other psychophysical tasks [44], [45] and similar to the one used by Gori and colleagues [46]) is as follows:
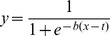
In this equation, x is the percentage of coherent motion in the display, y the relative response frequency.

The mean adjusted-R^2^ was .93±.03 (standard error of the mean or SEM. From now on, all the values after the sign ± represent the SEM value) across groups and condition (in particular for the central CDM: .93±.01, range: .87−.99, and .94±.01, range: .88−.99, for ASD and TD group, respectively; for the peripheral CDM: .94±.01, range: .88−.99, and .92±.02, range: .85−.99, for ASD and TD group, respectively).

Threshold is generally defined by an arbitrarily selected criterion resembling one point on the psychometric function at which the participant can detect the presence of, or a difference in, a stimulus with a given probability (e.g. [47]). The threshold of 75% accuracy in the motion direction discrimination was chosen as a reliable measure of good performance. A mixed design analysis of variance (ANOVA) was performed for the 75% thresholds in the CDM task, with group as between-subjects factor (ASD vs. TD), and condition as within-subjects factor (central and peripheral). A significant condition by group interaction was found (F_(1, 20)_ = 4.49, p<.05), indicating that CDM perception across the two conditions was different between the two groups. Between-subjects planned comparison revealed that the mean threshold was significantly higher in the ASD compared to the TD group in the central condition (.47±.08 and .27±.03 for ASD and TD group, respectively; t_(12.43)_ = 2.37, p<.05; Figure 1B), but not in the peripheral condition (.44±.05 and .44±.06 for ASD and TD group, respectively; t_(20)_ = 2.37, p>.05; Figure 1B). In addition, within-subjects planned comparison showed that mean threshold was lower in the central than the peripheral condition only for TD children (t_(10)_ = −3.15, p<.05).

### Attentional zooming task

Although the results of the attentional zooming task are extensively explained in the previous study [30], we analyze the primary results here because the group of children who took part in the previous research differed by one participant from the group of children participating in the present study.

Errors analysis revealed that the two groups did not differ in the amount of omission (mean ASD = 1%±.01, mean TD = 1%±.01; t_(20)_ = −.02, p>.05) and false alarms (i.e. response to catch trials; mean ASD = 5%±.03, mean TD = 5%±.02, t_(20)_ = −.14, p>.05). Also the d' between the two groups did not differ (mean ASD = 3.41±.36, mean TD = 3.75±.1; t_(20)_<1).

The attentional gradient effect (i.e., the difference in target RTs between 12 deg and 2 deg eccentricities) was calculated in the large and small cue condition. In the large cue condition, it is expected that the gradient effect will be lower (or absent), indicating efficient spread of attentional resources (zoom-out mechanism; see Figure 2C). In the small cue condition, however, the gradient effect should increase, indicating efficient focusing of attentional resources (zoom-in mechanism; see Figure 2D). These results are expected only when attentional zooming is present, which normally happens at short cue-target SOA (e.g., 100 ms), but not at longer SOA (e.g., 800 ms).

**Figure 3:**
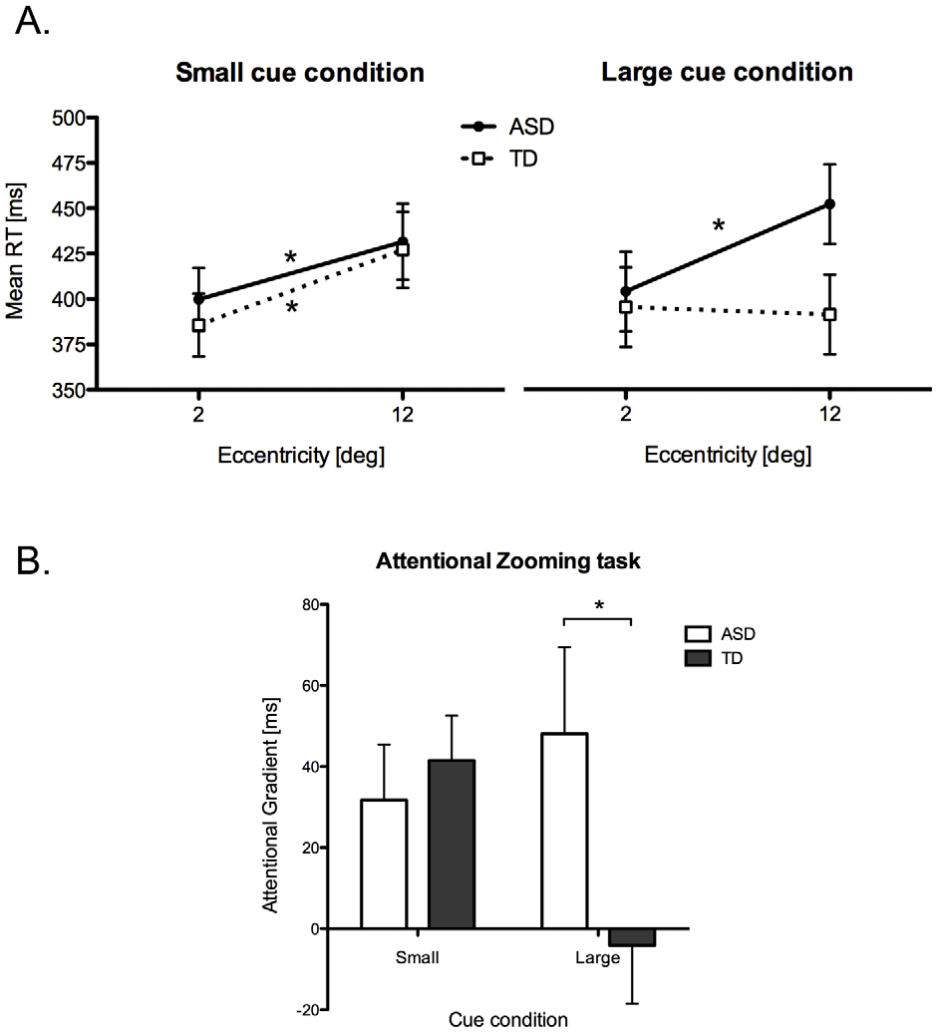
Results of the attentional zooming task showing: A) the mean reaction times as a function of cue size, eccentricity and group; B) the mean attentional gradient for ASD and TD groups as a function of cue size (large vs. small). Error bars represent the standard error of the mean and * indicates a significant difference revealed by planned comparisons.

At the first cue-target SOA (100 ms), the gradient effect was analyzed using a two-way ANOVA with the cue condition (small and large) as within-subjects factor, and group (ASD and TD) as between-subjects factor. Cue and group main effects were not significant. However, the cue by group interaction was significant (F_(1, 20)_ = 5.45, p<.05; see Figure 3). Planned comparisons revealed that the gradient effect was different in the two groups only in the large cue condition (t_(20)_ = 2.03, p<.05; mean ASD = 48.10±11, mean TD = −4.11±14), confirming a selective zoom-out deficit in children with ASD.

The same ANOVA at the second SOA (800 ms) did not shown any significant main effect or interaction (all ps>.05).

### Relationship between CDM performance and zoom-out attentional index

**Figure 4:**
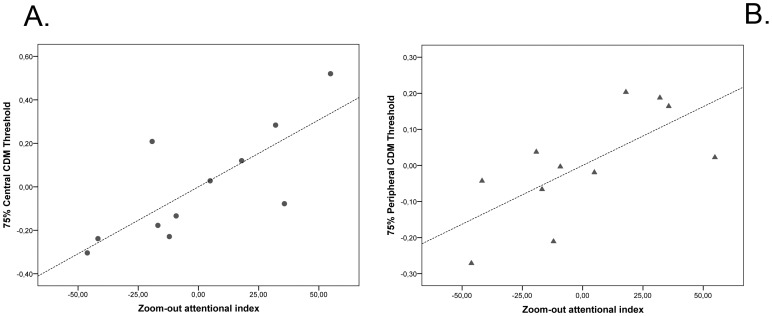
Partial correlation plot showing the relationship between central (Panel A) and peripheral (Panel B) CDM threshold and attentional gradient in large cue condition (i.e. zoom-out attentional index), controlled for age and performance IQ. Values on the x-axis represent the residuals of attentional zoom-out index. Values on the y-axis represent the residuals of threshold in the central CDM task.

In order to investigate our hypothesis, we studied the specific relationship between CDM (in the central and peripheral conditions) and the attentional zooming index. We used individual gradient effects in the large and small cue conditions, controlling for the effect of age and performance IQ. Partial correlation analyses in the ASD group revealed that the zoom-out attentional index (i.e. the gradient effects in the large cue condition) was positively related both to the central CDM threshold (r_(7)_ = .77, p<.05; Figure 4A) and to the peripheral CDM threshold (r_(7)_ = .69, p<.05; Figure 4B). This finding suggests the crucial role of the attentional zoom-out mechanism in children with ASD's CDM discrimination. In contrast, the individual zoom-in attentional index (i.e. the gradient effect in the small cue condition) was not related to the CDM discrimination in the ASD group (r_(7)_ = .28, p>.05 for the central CDM; r_(7)_ = .11, p>.05 for the peripheral CDM). In the TD participants, we did not find any significant relation between CDM and zoom-in or zoom-out index (all ps>.05).

Thus, only in the ASD group, we found a significant relation between the individual's ability to zoom-out the focus of attention and the CDM performance (in particular, in the ASD group a more severe attentional zoom-out disorder was related to higher CDM threshold; see Figure 4).

### Relationship between CDM task and autism symptom severity

**Figure 5:**
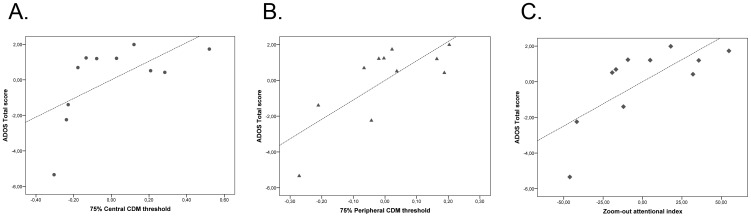
Partial correlation plots showing the relationship between the following: A) the threshold in the central CDM task and ADOS total interaction score; B) the threshold in the peripheral CDM task and ADOS total interaction score; C) the attentional zoom-out index and ADOS total interaction score. Values on the x-axis represent the residuals of central CDM threshold (Panel A), of peripheral CDM threshold (Panel B) and of attentional zoom-out index (Panel C). Values on the y-axis represent the residuals of ADOS total interaction score.

We considered the possible relationship between the individual CDM accuracy and ADOS score, controlling for the effect of age and performance IQ in our sample of children affected by ASD. Partial correlation analyses revealed that individual threshold in the central CDM condition was positively related to the ADOS total score (r_(7)_ = .62, p<.05; see Figure 5A). Similarly, the threshold in the peripheral condition was positively related to the ADOS total score (r_(7)_ = .75, p<.05; see Figure 5B).

### Relationship between zoom-out attentional index and autism symptom severity

We found a relationship between the zoom-out attentional index and ADOS total score (r_(7)_ = .73, p<.05; see Figure 5C). These results suggest that both the central-peripheral CDM accuracy and the efficiency in zooming-out the focus of attention were related to the severity of autism symptoms.

## Discussion

The debate on the coherent motion processing deficit in ASD is still open. Some authors suggest a general M-D stream vulnerability [8], although their results could be interpreted as a perceptual noise exclusion deficit [19]. Other evidence supports the hypothesis of a general difficulty in integrating the “global” signal [16], [17].

It has been demonstrated that the ability to discriminate integrative motion signals is better when stimuli are displayed in the central region of the visual field [31]–[33]. Accordingly, TD children revealed more efficient performance when dots were presented in the central compared to the peripheral condition.

An M-D deficit in children with ASD should predict a generally poorer performance in both conditions. However, children with ASD did not show a general impairment in the CDM task; rather, they presented only a specific deficit in the central condition compared to the TD children. Our group sizes were quite small, which could be considered a limitation; however it was large enough to show a significant difference in the central condition of the CDM task.

Our results support previous reports claiming that individuals with ASD present intact, low-level M-D stream processing, but an impaired high-level motion integration mechanism [16], [18], [48]. Moreover, the present results rule out the perceptual noise exclusion deficit interpretation. There is no theoretical reason to expect that the noise exclusion deficit will affect only the central condition.

According to the attentional zooming-out hypothesis, in the peripheral CDM condition, children with ASD were forced to use a large attentional focus to perform the task because of the complete absence of task relevant information in the central visual field. This ‘strategy’ maybe increased their motion integration. In the central CDM condition, however, children with ASD employed a narrow focus of attention, resulting in a worse performance (even if still fairly above chance level) compared to the TD children. Our correlation analyses support this hypothesis, showing a relationship between attentional zoom-out skills and CDM performance, even after controlling for chronological age and performance IQ. Thus, in the ASD group poorer ability to enlarge the spotlight of visual attention (zoom-out attentional deficit) was related to higher threshold in detecting the direction of coherently moving dots.

The present findings agree with the “weak central coherence” prediction [49], which proposes a failure in ASD individual's integration of visual stimuli, probably due to a superiority in local processing. However, a more direct link could be traced between our results and the “enhanced perceptual functioning” model (EPF) [50], [51].

The EPF model claims the superiority of low-level visual mechanisms in ASD that lead to an unbalanced relationship between high- and low-order visual processes. High-level attentional processes, previously demonstrated to have a role in coherent motion perception [21], [24], could not be recruited efficiently by observers with ASD.

This speculation seems to be confirmed by a dysfunction in the connectivity between top-down fronto-parietal attentional network and striate/extra-striate visual areas in ASD [52]–[54]. It seems that in individuals with ASD, the fronto-parietal attentional network does not “talk” properly with lower visual areas to perform the visual integration required in the CDM task. Preserved peripheral CDM perception suggests that spatio-temporal visual integration deficit could be reduced when ASD children are encouraged to explore the peripheral visual field. This last result, if confirmed in future studies, could open up possible strategies in visual rehabilitation for individuals with ASD.

## Conclusions

Our results show that a general M-D stream or a perceptual noise exclusion deficit in ASD are not exhaustive explanations for the decreased performance in CDM discrimination. On the other hand, decreased motion discrimination in children with ASD may result from an attentional inability to integrate the spatio-temporal information of coherently moving dots. We propose that participants with ASD fail to integrate coherent motion signal because of their reduced attentional zoom out abilities. However, it seems that this deficit could be partially overcome when no task-relevant information was displayed in the central portion of the visual field (i.e., CDM peripheral condition).

As suggested by the significant relation we found between zoom-out ability, CDM discrimination and autism severity, the attentional zoom-out deficit could produce a cascade effect on higher domains, such as communication and social abilities.
